# Akzeptanz und Nutzung von E-Mental-Health-Angeboten unter Studierenden: Sekundäranalyse eines Experiments

**DOI:** 10.1007/s11553-022-00945-1

**Published:** 2022-04-25

**Authors:** Jennifer Apolinário-Hagen, Mathias Harrer, Christel Salewski, Dirk Lehr, David Daniel Ebert

**Affiliations:** 1 Medizinische Fakultät, Institut für Arbeits‑, Sozial- und Umweltmedizin, Centre for Health and Society, Heinrich-Heine-Universität Düsseldorf, Düsseldorf, Deutschland; 2 Institut für Psychologie, Lehrstuhl für Klinische Psychologie und Psychotherapie, Friedrich-Alexander-Universität Erlangen-Nürnberg, Erlangen, Deutschland; 3 Fakultät für Psychologie, Lehrgebiet Gesundheitspsychologie, FernUniversität in Hagen, Hagen, Deutschland; 4 Institut für Psychologie, Gesundheitspsychologie und Angewandte Biologische Psychologie, Leuphana Universität Lüneburg, Lüneburg, Deutschland; 5 Fakultät für Sport- und Gesundheitswissenschaften, Professur für Psychology & Digital Mental Health Care, Technische Universität München, München, Deutschland

**Keywords:** E‑Health, Psychische Gesundheit, Technologieakzeptanz, Innovationsdiffusion, Studierende, Befragung, E‑health, Mental health, Technology acceptance, Diffusion of innovations, Students, Survey

## Abstract

**Zielsetzung:**

Trotz der Effektivität verschiedener E‑Mental-Health-Interventionen wurden bislang verhältnismäßig geringe Nutzungsraten, selbst unter Digital Natives wie Studierenden, identifiziert. Ziel dieser Studie ist daher, das Verhältnis der generellen Akzeptanz, dem konkreten Interesse und der tatsächlichen Registrierung für ausgewählte, zielgruppenspezifische E‑Mental-Health-Programme zu untersuchen.

**Methodik:**

Wir führten eine Sekundäranalyse einer in ein Online-Experiment eingebetteten Befragungsstudie mit *n* = 451 Studierenden (89 % Fernstudierende) zu Informationseffekten auf die Akzeptanz von E‑Mental-Health-Angeboten mit Untersuchung der Nutzungsabsicht sowie des Interesses im Verhältnis zu objektiven Daten, d. h. Registrierungen für ausgewählte E‑Mental-Health-Angebote zur Stressprävention und Gesundheitsförderung, durch.

**Ergebnisse:**

Eine hierarchische Regressionsanalyse ergab das Stresslevel, wahrgenommene Ähnlichkeit mit Informationsquellen und Einstellungen als Determinanten der Nutzungsabsicht (*R*^2^ = 0,49). Aktuelles Interesse an der Teilnahme an einem bestimmten E‑Mental-Health-Angebot berichtete weniger als ein Drittel der Stichprobe (31 %). Überdies war die Intentions-Verhaltens-Lücke bei der Follow-up-Messung (*n*/*N* in %) beim Programm für Berufstätige geringer (85 % registriert) als für das Programm für Studierende (69 % registriert; insgesamt: 77 %).

**Schlussfolgerung:**

Über drei Viertel der interessierten Studierenden haben sich für ein Programm registriert, was für die Bereitstellung einfacher, direkter Zugangsoptionen spricht. Zukünftige Studien sollten die Determinanten der Nutzung sowie Adhärenz bei E‑Mental-Health-Angeboten in Abhängigkeit von der Akzeptanz für verschiedene Subgruppen von Studierenden zur Entwicklung passgenauer Akzeptanzförderungsmaßnahmen genauer untersuchen.

**Zusatzmaterial online:**

In der Online-Version dieses Artikels 10.1007/s11553-022-00945-1 finden Sie zusätzliches Material

## Abstract (en)

**Objective:**

Despite the effectiveness of various e‑mental health interventions, relatively low usage rates have been identified, even among digital natives like university students. The aim of this study is therefore to examine the relationship between the general acceptance, the specific interest and actual registration rates for selected targeted e‑mental health programs.

**Methods:**

We conducted a secondary analysis of a survey study with *n* = 451 students (89% distance-learning students) that was embedded in an online experiment targeting information effects on the acceptance of e‑mental health services, including the investigation of usage intentions and interest in relation to objective data in terms of registrations regarding selected e‑mental health services for stress prevention and health promotion.

**Results:**

A hierarchical regression analysis showed the stress level, perceived similarity with information sources and attitudes as determinants of usage intentions (*R*^2^ = 0.49). Less than one-third of the sample (31%) reported current interest in participating in one specific e‑mental health service. Furthermore, the intention–behavior gap at the follow-up assessment (*n*/*N* in %) was lower for the program for employees (85% registered) than for the program for students (69% registered; 77% overall).

**Conclusion:**

More than three quarters of interested students have registered for a program, which calls for providing simple, direct access options. Future studies should examine the determinants of use and adherence to e‑mental health services depending on the acceptance in various subgroups of students in order to develop tailored acceptance-facilitating measures.

## Einleitung

Digitale Gesundheitsangebote halten zunehmend Einzug ins deutsche Gesundheitswesen, zuletzt befördert durch die COVID-19-Pandemie („coronavirus disease 2019“; [15]). Gerade digitalisierte psychologische Angebote („e-mental health services“; eMHSs) bieten verbesserte Chancen für die Versorgung und Prävention psychischer Störungen [23, 26]. Solche eMHSs umfassen ein breites Spektrum an digitalen Interventionen, von Stressmanagement-Apps bis hin zur Psychotherapie via Videokonferenz, die den Zugang zu Gesundheitsangeboten erleichtern und bestimmte Zielgruppen besser erreichen könnten als Face-to-face-Angebote alleine [13]. Hierzu zählen Studierende [12], die zwar einen erhöhten Bedarf an Unterstützung aufweisen, aber zugleich seltener professionelle Hilfe als andere Bevölkerungsgruppen in Anspruch nehmen [5]. Als Gründe hierfür berichten Studierende u. a. die Sorge vor einer Stigmatisierung und eine grundsätzliche Präferenz für Selbstbestimmung bei psychologischen Belangen [12]. Entsprechend eignen sich flexibel sowie anonym nutzbare eMHSs z. B. zur Stressbewältigung speziell für Studierende. Als eine Gruppe mit besonderem Belastungsprofil sind hier Fernstudierende hervorzuheben, die oft Mehrfachbelastungen erleben (z. B. Vereinbarkeit von Berufstätigkeit und Studium) und daher über weniger zeitliche Flexibilität verfügen [1, 17].`

Obwohl die Forschungslage die Wirksamkeit qualitätsgeprüfter eMHSs bei einer Reihe an psychologischen Indikationen sowie zur Gesundheitsförderung bestätigt [7, 13], bleibt die Nutzung bislang allerdings gering [14], selbst unter Digital Natives wie Studierenden [22]. Mögliche Gründe dafür umfassen die mangelnde Bekanntheit evidenzbasierter eMHSs sowie Unsicherheiten bei deren Beurteilung [9]. Im Fall der Notwendigkeit einer Intervention könnte dies letztlich die Entscheidung für die bekannteren Face-to-face-Angebote begünstigen [8, 23]. Daher erscheinen Informationsmaßnahmen als sinnvoller Ansatz zur Erhöhung des Bekanntheitsgrads von qualitätsgeprüften eMHSs. So zeigt aktuelle Forschung eine akzeptanzfördernde Wirkung von psychoedukativen Informationen über eMHSs [2, 11, 29].

Allerdings ist derzeit unklar, wie genau solche Informationen aufbereitet sein sollten, um die Bekanntheit und die Nutzung von eMHSs bei Studierenden zu erhöhen.

Ein vielversprechender Weg zur Steigerung der Akzeptanz von eMHSs sind Testimonials. Dies sind wertende Aussagen über einen Gegenstand, die einer Person zugeschrieben werden, die z. B. aufgrund von wahrgenommener Ähnlichkeit als besonders glaubwürdig angesehen wird. Daher stellen Testimonials eine alltägliche, allgemein leicht verständliche Form der Gesundheitskommunikation dar [28]. Studien zu Testimonialeffekten bei Gesundheitsentscheidungen deuten darauf hin, dass solche narrative Informationen Zielgruppen ohne Vorerfahrung leichter erreichen könnten als rein faktenbasierte, statistische Informationen [27]. Eine Kombination von Informationen zu bestimmten eMHSs mit persönlichen Erfahrungsberichten könnte demnach einen Ansatz zur ansprechenderen Gestaltung der Informationen sein, gerade wenn es an Vorwissen, Interesse oder der aktuellen Motivation zur gründlichen Auseinandersetzung mit solchen Informationen [27] sowie eigener Behandlungserfahrung mangelt [24]. Für die Wirksamkeit von Testimonials scheinen eine Reihe von Determinanten bedeutsam zu sein, wie z. B. längere und auf die Zielgruppe zugeschnittene Statements, höhere wahrgenommene Ähnlichkeit sowie Vertrauens- und Glaubwürdigkeit [16, 27, 28].

Bislang erbrachten Studien zu Testimonials, die sich häufig auf hypothetische somatische Behandlungs- oder Screening-Entscheidungen beziehen, jedoch inkonsistente Befunde [6, 28]. Zudem ist die Studienlage für die Nützlichkeit von Testimonials als Entscheidungshilfen im Bereich psychischer Gesundheit sehr limitiert [2, 29], insbesondere in Bezug auf die Erforschung der Effekte von potenziell akzeptanzfördernden Informationsstrategien auf das tatsächliche Verhalten, wie z. B. eine Registrierung oder die Nutzung von bestimmten, evidenzbasierten eMHSs [18]. Es existieren überdies bisher kaum Erkenntnisse zur Gestaltung und zum Einsatz von Testimonials als Methode zur Erhöhung der Akzeptanz von eMHSs bei Studierenden.

**Abb. 1:**
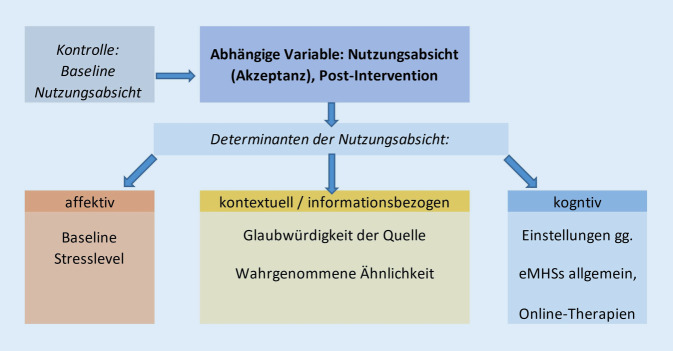
Konzeptuelles Modell der Nutzungsabsicht und ihrer Determinanten. *eMHSs* „e-mental health services“

Das übergeordnete Ziel der vorliegenden Studie ist daher die Gewinnung von ersten Erkenntnissen zur optimalen Gestaltung von Informationen zur Steigerung der Akzeptanz von eMHSs. Im Speziellen sollen Testimonials hinsichtlich ihrer Wirkung auf die Akzeptanz und Nutzung von eMHSs bei (Fern‑)Studierenden untersucht werden (Abb. 1).

### Zielsetzung und Forschungsfragen

Hierfür werden die Daten eines Online-Experiments zur Untersuchung akzeptanzfördernder Determinanten für die Nutzung von eMHSs [4] einer Sekundäranalyse unterzogen. Im Einzelnen werden dabei Einflussfaktoren auf die Nutzungsabsicht, das konkrete Interesse sowie die tatsächlichen Registrierungsraten für ausgewählte eMHSs zur Stressbewältigung sowie zum Aufbau von Resilienz betrachtet. Die Analyse wird von folgenden drei Forschungsfragen (FF) geleitet:

#### FF1.

Wie wirken sich das aktuelle Stresslevel, die wahrgenommene Ähnlichkeit mit den Testimonialquellen, deren Glaubwürdigkeit sowie die Einstellungen zu eMHSs auf die Nutzungsabsicht von eMHSs aus?

Bei den folgenden Forschungsfragen wurde zusätzlich die Zielgruppenpassung der eMHSs einbezogen. Speziell sollten hier mögliche Unterschiede in Akzeptanz und Nutzungsabsicht von eMHSs für Studierende eMHSs für Berufstätige adressiert werden.

#### FF2.

Welche Unterschiede gibt es bezüglich des Anteils an Studierenden mit Interesse an der Nutzung bestimmter eMHSs vs. ohne Interesse an der Nutzung in Abhängigkeit von der dargestellten Informationsart (FF2a) oder dem aktuellen Stresslevel (FF2b)?

#### FF3.

Wie ist das Verhältnis zwischen dem Interesse an der Inanspruchnahme und den tatsächlichen Anmeldungsraten bei eMHSs für Studierende vs. eMHSs für Berufstätige?

## Methodik

### Studiendesign und Setting der Primärstudie

**Abb. 2:**
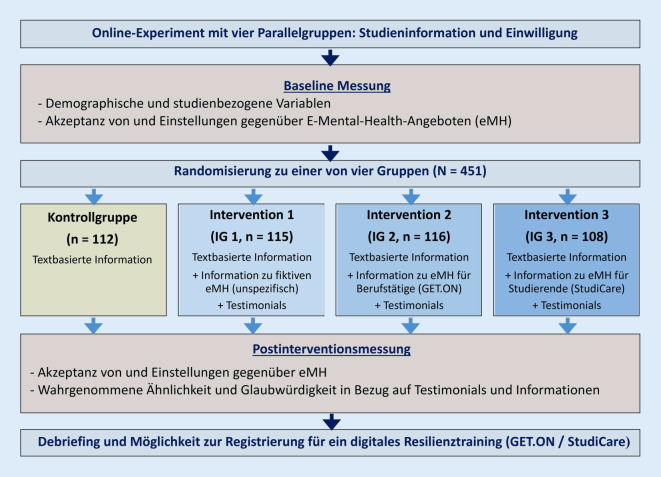
Studienablauf der Primärstudie

Die dieser Sekundäranalyse zugrunde liegende Primärstudie wurde als anonymisiertes Online-Experiment mit vier Studienarmen und einem Pre-Post-Design realisiert (für eine ausführliche Beschreibung: [4]). Die Studie wurde zwischen November 2018 bis Mai 2019 im Einklang mit der Deklaration von Helsinki in der aktuellen Fassung durchgeführt und zuvor vom Rektoratsbeauftragten der Fernuniversität in Hagen für die ethische Beurteilung von Forschungsprojekten im Herbst 2018 als unbedenklich eingestuft. Zusätzlich wurde diese Bewertung durch die Ethikkommission der Medizinischen Fakultät der Heinrich-Heine-Universität Düsseldorf nach der Datenerhebung bestätigt. Eingeschlossen wurden Studierende über 18 Jahre, die überwiegend an einer staatlichen Fernuniversität studierten. Ziel war die Analyse der Wirkung verschiedener Arten von Informationsinhalten auf die Nutzungsabsicht von eMHSs. Die Studienarme bestanden aus (1) einer aktiven Kontrollgruppe („nur Informationen“) und drei Interventionsgruppen (IG), die zusätzliche Informationen plus jeweils drei verschiedene Testimonialarten erhielten: von (2) vage beschriebenen Nutzern zu einer unspezifischen, hypothetischen eMHS (IG1), (3) von Berufstätigen zu *GET.ON*-Trainings (IG2) oder (4) von (Fern‑)Studierenden zu *StudiCare*-Trainings (IG3, s. Abb. 2).

### Durchführung und Messinstrumente der Primärstudie

Nach einer Baseline-Messung mit Fragen zur Person, Erfahrung mit eMHSs, wahrgenommenem Stress, Nutzungsabsichten und Einstellungen zu eMHSs wurden die Teilnehmenden randomisiert einer der vier Gruppen zugeteilt (vgl. [4]) und mit den vier verschiedenen Informationsinhalten konfrontiert (s. Online-Supplement). Der wahrgenommene Stress in den letzten 2 Wochen wurde mit der *Perceived Stress Scale* (PSS-10; [20], 10 Items, 5‑stufige Likert-Skala von 1 „nie“ bis 5 „sehr oft“ gemessen [α = 0,89]). Die Nutzungsabsicht wurde bei der Baseline- (α = 0,74) und Post-Interventionsmessung (α = 0,74–0,82) anhand von 3 Items pro Zeitpunkt (7-stufige Likert-Skala von 1 „stimme überhaupt nicht zu“ bis 7 „stimme voll und ganz zu“) basierend auf der „unified theory of acceptance and use of technology“ (UTAUT; [30]) und deren Anpassung auf eMHSs [11, 19] bestimmt. Einstellungen gegenüber eMHSs zur Gesundheitsförderung wurden ebenfalls mit einer 7‑stufigen Kurzskala zur Prä- und Post-Messung von Einstellungen zu eMHSs zur Gesundheitsförderung [4] ermittelt (Prä-Messung: α = 0,87; Post-Messung: α = 0,77–0,88).

Anschließend wurden bei der Post-Intervention die Quellenglaubwürdigkeit (4 Items, α = 0,78–0,86) sowie bei den Teilnehmenden der drei IG die wahrgenommene Ähnlichkeit mit den Testimonialquellen (5 Items, α = 0,86–0,89) jeweils auf einer 7‑stufigen Likert-Skala von 1 „stimme überhaupt nicht“ bis 7 „stimme voll und ganz zu“ erfasst [4] und erneut von allen Teilnehmenden die Fragebögen zur Einstellung sowie zur Nutzungsabsicht ausgefüllt. Zusätzlich wurden Einstellungen gegenüber Online-Therapien über die Fragebögen „attitudes towards psychological online interventions“ (APOI, [26]; 16 Items, α = 0,83) und „e-therapy attitudes measure“ (ETAM; [3]; 17 Items, α = 0,87), jeweils mit 5‑stufiger Antwortskalierung, erhoben. Nach dem Debriefing wurde das Interesse an der kostenlosen Teilnahme an einem digitalen Resilienztraining per Forced-choice-Format (d. h. Interesse an *StudiCare*, Interesse an *GET.ON*, generell kein Interesse, aktuell kein Interesse, unentschlossen) abgefragt. Im Falle von Interesse an einem der Programme folgte automatisch ein Link zur Registrierungswebsite, die nur die zuvor gewählte Option zuließ. Die Anmeldezahlen wurden bis zum letzten Tag der Befragung protokolliert.

### Statistische Analysen der Sekundäranalyse

Alle Analysen wurden mit SPSS^®^ Statistics, Version 25.0 (IBM Corp., Armonk, NY, USA), ausgeführt (Irrtumswahrscheinlichkeit: α < 0,05). Eine hierarchische Regressionsanalyse wurde durchgeführt, um die relativen Beiträge des Stresslevels, der wahrgenommenen Ähnlichkeit, Glaubwürdigkeit der Quelle (Schritt 1) und Einstellungen gegenüber eMHSs (Schritt 2) bei der Varianzaufklärung bei den Nutzungsabsichten von eMHSs zu bestimmen. Im 3. Schritt wurde die Baseline-Nutzungsabsicht als Kontrollvariable einbezogen (FF1). Unterschiede zwischen den vier Versuchsgruppen (KG, IG1-3) beim Interesse an eMHSs wurden per χ^2^-Test nach Pearson (FF2a) und zwischen den Interessengruppen beim wahrgenommenen Stress per einfaktorieller Varianzanalyse exploriert (FF2b). Die Bewertung des Verhältnisses des Interesses zu den Registrierungsraten erfolgte deskriptiv (FF3).

## Ergebnisse

### Deskriptive Daten und Voranalysen

Die Charakteristika der Stichprobe sind in Tab. 1 und Tab. S1 im Online-Supplement aufgelistet.

**Tab. 1:** Merkmale der Stichprobe (*n* = 451)

Variablen		Anteil, *n* (% von *n* = 451)
*Geschlecht*	Weiblich	340 (75,4)
	Männlich	110 (24,4)
	Anderes/divers	1 (0,2)
*Alter (Jahre)*	M (SD), Range (Jahre)	32,6 (10,29), 18–65
*Bildungsabschluss*	Fachoberschulreife oder Meisterbrief	23 (5,1)
	Fach- oder Allgemeine Hochschulreife	216 (47,9)
	Hochschulabschluss	173 (38,3)
	Sonstige	39 (8,6)
*Studienprogramm/Hochschulart*	Fernstudium/Fernuniversität	400 (88,7)
	Präsenzstudium/Präsenzhochschule^a^	27 (6,0)
	Kombination aus Präsenz- und Fernstudium	23 (5,1)
	Anderes	1 (0,2)
*Vertrautheit/Bekanntheit von eMHSs*^b^	Nein	224 (49,7)
	Ja	191 (42,4)
	Weiß nicht	36 (8,0)
*Informationen über eMHSs gesucht*^c^	Nein	142 (31,5)
	Ja	75 (16,6)
	Weiß nicht	10 (2,2)
*Erfahrung mit der Nutzung von eMHSs*^d^	Nein	183 (40,6)
	Ja	32 (7,1)
	Weiß nicht	12 (2,7)

*M* Mittelwert, *SD* Standardabweichung, *eMHSs* „e-mental health services“

^a^Traditionelle Präsenzhochschule in Abgrenzung zu Fernhochschulen (Durchführung der Studie von November 2018 bis Mai 2019, d. h. vor dem Ausbruch der COVID-19-Pandemie [„coronavirus disease 2019“] in Deutschland)

^b^„Haben Sie schon einmal vor dieser Studie von E‑Mental-Health-Angeboten gehört?“

^c^„Haben Sie sich schon zu einem oder mehreren E‑Mental-Health-Angeboten näher informiert?“

^d^„Haben Sie schon ein oder mehrere E‑Mental-Health-Angebote genutzt?“

Da die unterschiedlichen Informationen keinen Einfluss auf die Nutzungsabsichten bei der Post-Messung zwischen den vier Versuchsgruppen aufwiesen (*F*_[3,447]_ = 1,45, *p* = 0,227, *ŋ*_p_^2^ = 0,01), wurden diese in der Sekundäranalyse nicht näher betrachtet. Unterschiede fanden sich hingegen bei den Determinanten Einstellung gemäß APOI, Glaubwürdigkeit und wahrgenommener Ähnlichkeit zugunsten zielgruppenspezifischer Informationen (s. Tab. S2 im Online-Supplement).

#### (FF1) Determinanten der Nutzungsabsicht

Im 2. Block des Regressionsmodells hatten das Stresslevel, die wahrgenommene Ähnlichkeit und Einstellungen gegenüber eMHSs (∆*R*^2^ = 0,26 von *R*^2^ = 0,25 auf *R*^2^ = 0,49) einen signifikanten Einfluss auf die Nutzungsabsicht bei Post-Messung (*p*_s_ < 0,001). Glaubwürdigkeit war nur in Block 1 signifikant. Nach Hinzufügung der Baseline-Werte der Nutzungsabsicht stieg *R*^2^ weiter von 49 % (Block 2) auf 74 % (Block 3), während das Stresslevel und die Einstellung im APOI nicht signifikant wurden (s. Tab. 2).

**Tab. 2:** Hierarchische Regressionsanalyse der Determinanten der Nutzungsabsichten bezüglich eMHSs („e-mental health services“) unter Studierenden (*n* = 451)

**Block 1**	**Variable**	**B**	***SE* (B)**	**β**	**T**	***p***	**95** **%-KI (B)**		***R***^**2**^
–	(Konstante)	0,004	0,361	–	0,012	0,990	−0,706	0,714	–
1	*Stresslevel*	0,207	0,081	0,109	2,547	0,011	0,047	0,367	*R*^2^ = 0,26
2	*Glaubwürdigkeit*	0,430	0,051	0,343	8,377	< 0,001	0,329	0,531	
3	*Wahrgenomme Ähnlichkeit*	0,390	0,055	0,302	7,062	< 0,001	0,282	0,499	
**Block 2**	**Variable**	**B**	***SE* (B)**	**β**	**T**	***p***	**95** **% KI (B) Step**		***R***^**2**^
–	(Konstante)	−1,274	0,353	–	−3,605	< 0,001	−1,968	−0,579	–
1	Stresslevel	0,161	0,068	0,085	2,369	0,018	0,027	0,294	*R*^2^ = 0,26
2	Glaubwürdigkeit	0,049	0,050	0,039	0,981	0,327	−0,050	0,148	
3	Wahrgenomme Ähnlichkeit	0,261	0,047	0,202	5,583	< 0,001	0,169	0,353	
4	*Einstellung E‑Mental-Health*	0,532	0,056	0,480	9,535	< 0,001	0,423	0,642	*R*^2^ = 0,49 *∆R*^*2*^ = 0,25
5	*Einstellung Online-Therapie (APOI)*	0,301	0,142	0,125	2,115	0,035	0,021	0,581	
6	*Einstellung Online-Therapie (ETAM)*	0,052	0,123	0,023	0,418	0,676	−0,191	0,294	
**Block 3**	**Variable**	**B**	***SE* (B)**	**β**	**T**	***p***	**95** **% KI (B) Step Step 2**		***R***^**2**^
–	(Konstante)	−1,378	0,254	–	−5,418	< 0,001	−1,878	−0,878	–
1	Stresslevel	0,049	0,049	0,026	0,989	0,323	−0,048	0,145	*R*^2^ = 0,26
2	Glaubwürdigkeit	0,063	0,036	0,051	1,751	0,081	−0,008	0,135	
3	Wahrgenomme Ähnlichkeit	0,192	0,034	0,149	5,679	< 0,001	0,126	0,259	
4	Einstellung E‑Mental-Health	0,224	0,043	0,202	5,227	< 0,001	0,140	0,309	*R*^2^ = 0,49 *∆R*^*2*^ = 0,25
*5*	Einstellung Online-Therapie (APOI)	0,038	0,103	0,016	0,367	0,714	−0,165	0,241	
*6*	Einstellung Online-Therapie (ETAM)	0,002	0,089	0,001	0,024	0,981	−0,172	0,177	
*7*	*Baseline-Nutzungsabsicht*	0,713	0,035	0,635	20,354	< 0,001	0,645	0,782	*R*^2^ = 0,74 *∆R*^*2*^ = 0,26

*SE* „standard error“ B, *KI* Konfidenzintervall, *B* unstandardisierter Koeffizient, *β* standardisierter Koeffizient (Beta-Gewicht)

Abhängige Variable: Nutzungsabsicht bezüglich eMHSs („e-mental health services“, Post-Intervention)

Aufgeklärte Varianz: Block 1: *R*^2^ = 0,26, korrigiertes *R*^2^ = 0,25, *F*_(3,447)_ = 50,96 (*p* < 0,001), Block 2: *R*^2^ = 0,49, korrigiertes *R*^2^ = 0,48, *F*_(3,444)_ = 68,14 (*p* < 0,001), ∆*R*^2^ = 0,26, Block 3: *R*^2^ = 0,74, korrigiertes *R*^2^ = 0,73, *F*_(1,443)_ = 414,30 (*p* < 0,001), ∆*R*^2^ = 0,25. Die fehlenden Werte bei der Variable „Wahrgenommene Ähnlichkeit“ und teilweise auch bei der Variable „Glaubwürdigkeit“ (2 von 4 Items zur Glaubwürdigkeit von Testimonials) wurden bei der Kontrollgruppe durch den Mittelwert ersetzt

#### (FF2a) Unterschiede im Interesse an eMHSs

Insgesamt interessierten sich 16 % (*n* = 72) für *StudiCare* und 13,3 % (*n* = 60) für *GET.ON *(Tab. 3). Die meisten Studierenden waren derzeit (*n* = 151, 33,6 %) oder allgemein (*n* = 68, 15,1 %) nicht an eHMSs interessiert, während die restlichen unentschlossen waren (*n* = 99, 22 %).

**Tab. 3:** Interesse an eMHSs innerhalb der vier Versuchsgruppen (*n* = 450)^a^

Interesse Rating („forced choice“)		Generell kein Interesse an GET.ON oder StudiCare	Aktuell kein Interesse an GET.ON oder StudiCare	Interesse an StudiCare Resilienz	Interesse an GET.ON RESIST	Unentschlossen	Gesamt (*n* [%])
Interesse innerhalb der IG (*n* [%])	KG	19 (4,2)	37 (8,2)	15 (3,3)	13 (2,9)	28 (6,2)	112 (24,9)
	IG1	18 (4,0)	39 (8,7)	14 (3,1)	20 (4,4)	24 (5,3)	115 (25,6)
	IG2	15 (3,3)	40 (8,9)	17 (3,8)	16 (3,6)	28 (6,2)	116 (25,8)
	IG3	16 (3,6)	35 (7,8)	26 (5,8)	11 (2,4)	19 (4,2)	107 (23,8)
*Interesse insgesamt (n [%])*		68 (15,1)	151 (33,6)	72 (16,0)	60 (13,3)	99 (22,0)	450 (100)

*KG* Kontrollgruppe (nur Informationen), *IG1* MH-Online-Testimonials zu unspezifischen, hypothetischen eMHSs („e-mental health services“) sowie Informationen, *IG2* GET.ON-Testimonials für Arbeitnehmer sowie Informationen, *IG3* StudiCare-Testimonials für Studierende sowie Informationen

^a^Fehlende Angabe in IG3 (*n* = 1)

Zwischen den vier Versuchsgruppen gab es keine signifikanten Unterschiede bezüglich des Interesses an Programmregistrierungen, χ^2^_(12,__*N*_ _=_ _450)_ = 11,05, *p* = 0,524.

#### (FF2b) Zusammenhänge zwischen Stress und Interesse an eMHSs

Eine einfaktorielle Varianzanalyse ergab einen signifikanten Unterschied beim wahrgenommenen Stress zwischen den fünf Interessengruppen (*F*_(4,445)_ = 4,47, *p* = 0,002, *ŋ*_p_^2^ = 0,04).

Tuckey-HSD-adjustierte Post-hoc-Tests zeigten bei Studierenden mit Interesse an *GET.ON* (*M* = 2,99, *SD* = 0,67) höhere Stresslevels bei der Baseline-Messung als bei denjenigen, die momentan (*M* = 2,61, *SD* = 0,59; M_diff_ = 0,38, *SE* = 0,10, *p* = 0,001, 95 % [0,12, 0,65]) oder allgemein (*M* = 2,58, *SD* = 0,64; M_diff_ = 0,41, *SE* = 0,12, *p* = 0,004, 95 % KI [0,09, 0,72]) kein Interesse an einem der eHMSs berichteten.

#### (FF3) Verhältnis von Interesse und Registrierungen

Obwohl mehr Studierende Interesse an *StudiCare* (*n* = 72) als an *GET.ON* (*n* = 60) angaben, war die Zahl der tatsächlichen Anmeldungen pro Training nahezu identisch (*n* = 50 für *StudiCare, n* = 51 für *GET.ON*). Insgesamt 76,5 % mit Interesse registrierten sich für eines der Trainings. Die Lücke zwischen beabsichtigter (*N*) und tatsächlicher (*n*) Registrierung beim Follow-up (*n*/*N* in %) war bei *GET.ON *geringer (*N* = 60 beabsichtigt, *n* = 51 registriert; 85,0 %) als für *StudiCare* (*N* = 72 beabsichtigt, *n* = 50 registriert; d. h. 69,4 %).

## Diskussion

Diese Sekundäranalyse untersuchte akzeptanzfördernde Faktoren von Informationsmaterialien bezüglich eMHSs unter Studierenden. Einflussfaktoren der Nutzungsabsicht umfassten in unserer Studie das Stresslevel, die wahrgenommene Ähnlichkeit mit Testimonialquellen sowie Einstellungen. Der positive Einfluss von Ähnlichkeit oder Identifikation mit Testimonialquellen auf Überzeugungen und Einstellungen entspricht früherer Forschung [16, 27, 28] und deutet auf einen potenziellen Nutzen von Informationsmaterial hin, das auf die spezielle Lebenswelt von Studierenden zugeschnitten wird. Wider Erwarten spielte die Glaubwürdigkeit der Informationen nur eine untergeordnete Rolle bei der Vorhersage der Nutzungsabsichten, was an der geringen Varianz gelegen haben mag. Die Glaubwürdigkeit von Informationen kann jedoch durch Testimonials zu eMHSs auch eingeschränkt werden [10]. In der vorliegenden Studie wurden nur positive Aussagen über eMHSs verwendet, was bei Nutzerbewertungen unrealistisch erscheinen mag. Infolgedessen sollte zukünftige Forschung die Valenz von Testimonials variieren [25].

Ein weiterer Schwerpunkt befasste sich mit Einflussfaktoren auf das Interesse an nichttherapeutischen eMHSs. Hier fanden sich keine Unterschiede zwischen den vier Versuchsgruppen bei den Interessenbekundungen. Möglicherweise waren die Unterschiede zwischen den dargestellten Interventionen zu gering und die Zeitspanne zwischen der Informationsdarbietung, der Entscheidung und der Registrierung nicht lang genug gewählt. Weiterhin wurde nicht erhoben, ob die Befragten andere digitale Selbsthilfetools (z. B. Meditations-Apps) nutzen oder anstelle der beschriebenen aufwendigeren, mehrwöchigen Trainings präferierten. Andere Studien legen hier eine Präferenz für schnelle Lösungen per eMHSs bei akutem Studienstress nahe [10].

In der vorliegenden Studie war das selbstberichtete Stresslevel der Teilnehmenden im Vergleich zur Allgemeinbevölkerung moderat erhöht [20], was einen Bedarf an Unterstützung im Besonderen auch bei Fernstudierenden bestätigt. Dies korrespondiert mit den in der internationalen Forschungsliteratur identifizierten, erheblichen Belastungen von Studierenden [5]. Daher erscheint es notwendig, sowohl für traditionelle als auch für nichttraditionelle, häufig berufstätige Studierende verschiedene Optionen zur Stressprävention bereitzustellen [17].

Ein vielversprechendes Ergebnis war, dass sich 23 % der Befragten (*n* = 101) für eines der beiden eMHSs registrierten, was über zwei Drittel (77 %) der Studierenden mit entsprechender Interessebekundung entspricht. Nahezu die gleiche Anzahl an Studierenden meldete sich für eines der beiden Trainings an. Es scheint daher grundsätzlich zielführend, einfache, direkt zugängliche Möglichkeiten zur Nutzung solcher eMHSs bereitzustellen. Darüber hinaus hat sich ein höherer Anteil an Studierenden mit Interesse an GET.ON (85 % von *n* = 60) im Vergleich zu StudiCare (69 % von *n* = 72) tatsächlich für dieses digitale Resilienztraining registriert, was auf eine kleinere Absicht-Verhaltens-Lücke hindeutet. Dieser Befund ist wahrscheinlich mit dem Überwiegen von Fernstudierenden in unserer Studie verbunden, die häufiger neben dem Studium berufstätig sind als traditionelle Studierende [1]. Die Gruppe mit Interesse an GET.ON zeigte zudem ein signifikant höheres Stresslevel als Befragte ohne Interesse. Da aus Datenschutzgründen die beiden Studienteile nicht miteinander verknüpft werden durften, lässt sich nur mutmaßen, dass die GET.ON-Interessierten eher berufstätige Studierende waren. Es wäre zukünftig wichtig, den Pfad vom Interesse über die Registrierung bis hin zur Nutzung (inklusive Adhärenz) im Alltag sowie den Erfolg der Teilnahme zukünftig genauer zu untersuchen, da die Studienlage hierzu noch begrenzt und inkonsistent ist [21]. Angesichts des kurzen Bewertungszeitraums ist es auch denkbar, dass Teilnehmende ohne aktuelles Nutzungsinteresse in der vorliegenden Studie später beschlossen, sich für diese oder andere eMHSs zu registrieren, weil die Studienteilnahme zu einer Sensibilisierung beigetragen hat. Dies könnte bedeuten, dass der wahre Effekt von Informationsinterventionen auf die Akzeptanz und Nutzung von eMHSs unterschätzt wurde.

### Limitationen

Die untersuchte Stichprobe bestand hauptsächlich aus Fernstudierenden, die sich häufig von traditionellen Studierenden in Aspekten wie persönlicher Hintergrund und Stressoren unterscheiden [1]. Dies schränkt die Generalisierbarkeit der Ergebnisse ein. Darüber hinaus wurden die Teilnehmenden nicht gefragt, ob sie sich eher mit Studierenden, Berufstätigen oder beidem identifizierten. Zudem war die Online-Studie möglicherweise recht textlastig, sodass dies einen verzerrenden Faktor darstellen kann (z. B. Rückgang der Aufmerksamkeit). Außerdem berichteten nur 7 % der Studierenden frühere Erfahrung mit eMHSs, was jedoch anderen Studien aus dem Erhebungszeitraum in Deutschland entspricht (z. B. [22]). Schließlich wurde nur allgemein nach Erfahrungen mit eMHSs gefragt, sodass keine Daten über Art oder Dauer der Nutzung vorliegen. Dadurch kann nicht ausgeschlossen werden, dass Teilnehmende ohne Interesse bereits eine andere Art von Unterstützung erhielten.

### Schlussfolgerung

Unsere Studie deutet darauf hin, dass die bloße Option der kostenlosen Registrierung im Zusammenhang mit Informationen zu evidenzbasierten eMHSs eine einfache und zugleich effiziente Strategie darstellen kann, um die Bekanntheit sowie Akzeptanz und womöglich auch Nutzung von qualitätsgeprüften Interventionen unter heterogenen Studierendengruppen zu erhöhen.

## Fazit für die Praxis

- Drei Viertel der befragten Studierenden mit bekundetem Interesse an einem E‑Mental-Health-Programm haben sich nach erstmaliger Information für das angebotene Programm registriert, was für die Bereitstellung einfacher, direkter Zugangsoptionen spricht.
- Zukünftige Studien sollten die Determinanten der Nutzung sowie Adhärenz bei E‑Mental-Health-Angeboten in Abhängigkeit von der Akzeptanz für verschiedene Studierendengruppen zur Entwicklung passgenauer Akzeptanzförderungsmaßnahmen untersuchen.

## Supplementary Information

Online-Supplement: Anhang 1: Methodenteil – Texte zu den Informationsinterventionen. Anhang 2: Methodenteil – Erhebungsinstrumente. Anhang 3: Ergänzende Tabellen aus dem Ergebnisteil.

## Abkürzungen

APOI: „Attitudes toward psychological online interventions“
EMHSs: „E-mental health services“
ETAM: „E-therapy attitudes measure“
FF: Forschungsfrage
PSS: „Perceived stress scale“
