# Supplementary material for: Acceptance and use of e-mental health services among university students: Secondary analysis of an experiment
Source: Pravent Gesundh. 2022 Apr 25;18(2):196–203. [Article in German] doi: 10.1007/s11553-022-00945-1 (PMC9037969; doi:10.1007/s11553-022-00945-1)
Supplement: Supplementary file 1 — Online-Supplement: Anhang 1: Methodenteil – Texte zu den Informationsinterventionen. Anhang 2: Methodenteil – Erhebungsinstrumente. Anhang 3: Ergänzende Tabellen aus dem Ergebnisteil. [file 11553_2022_945_MOESM1_ESM.docx]

Online Supplement

**Anhang 1: Methodenteil - Texte zu den Informationsinterventionen**

Allgemeiner Informationstext (alle Versuchsgruppen, d.h. KG, IG1, IG2, IG3)*:

Online-Gesundheitstrainings und -therapien (E-Mental-Health-Angebote) im Bereich der psychischen Gesundheit bieten neue Möglichkeiten zur Verbesserung bzw. Erweiterung des Angebots für Hilfesuchende. Psychische Erkrankungen werden oft nicht oder nicht ausreichend behandelt. Internetbasierte Psychotherapien (oder Internettherapien/Online-Therapien) werden als eine Möglichkeit angesehen, diese Lücke in der Versorgung zu schließen. Zahlreiche wissenschaftliche Studien belegen die Wirksamkeit internetbasierter Therapien bei einem breiten Spektrum psychischer Probleme, wie sie etwa durch dauerhaften Stress entstehen können. Es werden kognitiv-verhaltenstherapeutische Methoden eingesetzt, die auch in der klassischen Face-to-Face Therapie angewendet werden. Beispielsweise gibt es Trainings mit den Themen allgemeines Stressverhalten, leichte bis mittlere Depressionen, Angststörungen oder Essstörungen wie auch Stärkung von Resilienz. Eine mögliche Therapieform sind internetbasierte begleitete Programme, bei denen PatientInnen Online-Programme oder Online-Selbstlernmodule mit Unterstützung einer Therapeutin/ eines Therapeuten bearbeiten. Die Kommunikation mit der Therapeutin/dem Therapeuten erfolgt dabei per E-Mail, Chat oder Videokonferenz. Ein großer Vorteil für den Teilnehmenden ist hierbei die zeitliche sowie örtliche Flexibilität.

* Abkürzungen: KG=Kontrollgruppe, IG=Interventionsgruppe

Zusätzliche Information und Testimonials für IG1 (fiktiver Anbieter „MH-Online“):

MH-Online (Albert-Ludwigs-Universität Freiburg) bietet wissenschaftlich geprüfte und professionell therapeutisch begleitete Online-Trainings zum Beispiel in den Bereichen “Stress”, “Resilienz/Widerstandsfähigkeit” sowie “Depressionen/Ängste” an. Die Trainings bestehen aus 6 aufeinanderfolgenden Online-Einheiten mit jeweils circa einer Stunde Dauer. Begleitend zum Online-Training können Sie täglich eine App nutzen. Zudem besteht ergänzend die Möglichkeit eines persönlichen Coachings. Das Training zielt darauf ab, wieder zu mehr Leistungsfähigkeit zu gelangen. Beispiel: Im Stresstraining werden persönliche Themen identifiziert, Bewältigungsstrategien erprobt, Problemlösestrategien eingesetzt und der Umgang mit belastenden Gefühlen gelernt.

Teilnehmende, die die Online-Trainings von MH-Online genutzt haben, wurden nach ihren Erfahrungen gefragt:

**- „Die Online-Therapie hat mir geholfen. Das Programm und der Kontakt zu meinem Therapeuten haben gut funktioniert.“** (Petra Z., 35 Jahre)

**- „Meine Erfahrungen waren positiv. Ich würde MH-Online weiterempfehlen.“** (Sebastian L., 46 Jahre)

‑ **„Ich konnte meine Therapie mit MH-Online sehr gut in meinen Alltag integrieren. Mein Therapeut ist sehr gut auf mich eingegangen und hat mich immer unterstützt.“** (Jens R., 25 Jahre)

Zusätzliche Information und Testimonials für IG2 (realer Anbieter „GET.ON“):

Das GET.ON Institut (Leuphana Universität Lüneburg) bietet wissenschaftlich geprüfte und professionell therapeutisch begleitete Online-Trainings zum Beispiel in den Bereichen “Stress”, “Resilienz/Widerstandsfähigkeit” sowie “Depressionen/Ängste” an. Der Zugang ist dabei für eine Vielzahl an Personengruppen offen (z.B. Berufstätige, Auszubildende, Studenten, Rentner). Die Trainings bestehen aus 6 aufeinanderfolgenden Online-Einheiten mit jeweils circa einer Stunde Dauer. Begleitend zum Online-Training können Sie täglich eine

App nutzen. Zudem besteht ergänzend die Möglichkeit eines persönlichen Coachings. Das Training zielt darauf ab, wieder zu mehr Leistungsfähigkeit zu gelangen. Beispiel: Im Stresstraining werden persönliche Themen identifiziert, Bewältigungsstrategien erprobt, Problemlösestrategien eingesetzt und der Umgang mit belastenden Gefühlen gelernt.

Teilnehmende, die Online-Trainings von GET.ON genutzt haben, wurden nach ihren Erfahrungen gefragt:

**‑ "Den Stress auf der Arbeit habe ich nach und nach mit nach Hause genommen. Durch die wiederkehrenden Regeln im Training, stellte sich langsam ein Gewöhnungsprozess ein, so dass deren Einhaltung machbar war ohne zusätzlichen Stress aufzubauen."** (Klaus M., 51, Call-Center-Mitarbeiter, Stresstraining)"

**- Vor dem Training habe mich mit meinen beruflichen Problemen nur im Kreis gedreht. Insgesamt kann ich das Training allen empfehlen, die selbst an sich arbeiten wollen und die dazu das nötige Rüstzeug brauchen. Mich hat es innerhalb von Wochen viel stärker gemacht. Vielen Dank dafür!”** (Sandra F., 46, Angestellte, Problemlösetraining)

**- "Es hilft mir sehr, dass ich einige meiner Gedanken niederschreiben kann und von ihnen kommentiert bekomme. Natürlich machen ihre Antworten Mut, sich zum einen weiter mit sich selbst und den Problemen zu befassen und diese in den Griff zu bekommen. Zum anderen stoßen sie mich hier und da in eine neue Richtung, erwähnen Aspekte oder beschreiben Situationen aus einem etwas anderen Blickwinkel so dass sie mich zum Nachdenken anregen."** (Janine T., Lehrerin, 52 Jahre, Training Prävention depressiver Erschöpfung)

Zusätzliche Information und Testimonials für IG3 (realer Anbieter „StudiCare“):

StudiCare (Friedrich-Alexander-Universität Erlangen) bietet wissenschaftlich geprüfte und professionell therapeutisch begleitete Online-Trainings zum Beispiel in den Bereichen “Stress”, “Resilienz/Widerstandsfähigkeit” sowie “Depressionen/Ängste” speziell für Studierende an. Die Trainings bestehen aus 6 aufeinanderfolgenden Online-Einheiten mit jeweils circa einer Stunde Dauer. Begleitend zum Online-Training können Sie täglich eine App nutzen. Zudem besteht ergänzend die Möglichkeit eines persönlichen Coachings. Das Training zielt darauf ab, wieder zu mehr Leistungsfähigkeit zu gelangen. Beispiel:  Im Stresstraining werden persönliche Themen identifiziert, Bewältigungsstrategien erprobt, Problemlösestrategien eingesetzt und der Umgang mit belastenden Gefühlen gelernt.

Teilnehmende, die Online-Trainings von StudiCare genutzt haben, wurden nach ihren Erfahrungen gefragt:

**- „Es ist toll, dass ich als Fernstudentin jetzt auch Unterstützung für den Umgang mit mentalen Problemen bekomme. Ich habe besonders mit stressenden Prüfungsängsten zu tun. Da hat mir das Training von StudiCare und das Feedback meines Coaches sehr geholfen. Alleine schon zu wissen, dass da jemand ist, den ich anonym immer ansprechen kann, ist sehr beruhigend.”** (Anna S., Bachelor Kommunikationswissenschaften, 29, Training gegen Prüfungsangst)

**- „Am Anfang war ich etwas überrascht über die Art und Weise des Vorgehens. Schnell habe ich aber die Vorteile ähnlich dem Fernstudium genutzt. Als Einstiegstraining super. Hat mir geholfen mich dem Stress durch Doppelbelastung mit Job und Studium und den für mich dahinterstehenden Probleme zu stellen. Für mich der Einstieg in eine notwendige Psychotherapie face-to-face. Wäre ich ohne den „soften“ Einstieg nicht angegangen.“** (Tim K., Philosophie, 40, Training Prävention depressiver Erschöpfung)

**‑ „Ich konnte wo und wie es gepasst hat mitmachen. Das hat es mir überhaupt erst möglich gemacht neben FernStudium, Familie und Beruf an einem Training teilzunehmen - und es hat was gebracht! Empfehlenswert!“** (Claudia W, Master Psychologie, 37, Stresstraining)

**Anhang 2: Methodenteil - Erhebungsinstrumente**

Tabelle S1. *Übersicht über die Skalen und Items.*

| Skala/Variable | (Item-Nr.) Itemtext |
| --- | --- |
| Einstellung gg. eMHSs,  Kurzskala, Baseline | (1) Ich finde E-Mental-Health Angebote im Allgemeinen hilfreich.  (2) Ich finde E-Mental-Health Angebote können konventionelle Gesundheitsangebote sinnvoll unterstützen.  (3) Ich finde E-Mental-Health Angebote können konventionelle Gesundheitsangebote sinnvoll unterstützen. |
| Nutzungsabsicht -UTAUT  (Hennemann et al., 2016), Baseline | (1) Ich kann mir vorstellen, dass E-Mental-Health Angebote mein Wohlbefinden fördern können.  (2) Ich würde gerne ein E-Mental-Health Angebot ausprobieren.  (3) Ich plane, ein E-Mental-Health Angebot in den nächsten 3 Monaten zu nutzen. |
| Wahrgenommener Stress – PSS-10 (Cohen et al., 1983, Klein et al. 2016), Baseline | *Siehe: Klein et al. (2016)* |
| Wahrgenommene Ähnlichkeit,  Post-Intervention (nur IGs) | (1) Der Hintergrund der Personen ist ähnlich wie meiner.  (2) Die Personen haben eine ähnliche Bildung wie ich.  (3) Die Personen haben ähnliche Belastungen wie ich.  (4) Ich fühle mich in ähnlicher Weise betroffen wie die Personen.  (5) Die Lebenssituation der Personen ist ähnlich wie meine. |
| Glaubwürdigkeit der Quelle,  Post-Intervention (KG: Items 1+2, IGs: Items 1-4) | (1) Bei den dargebotenen Informationen war es in der Regel deutlich zu erkennen, ob es sich um Fakten oder um Meinungen handelte.  (2) Bei den Aussagen der Testimonials war es in der Regel deutlich zu erkennen, ob es sich um Fakten oder um Meinungen handelte.  (3) Die Angaben der Testimonials fand ich glaubwürdig.  (4) Die dargebotenen Informationen fand ich glaubwürdig. |
| Einstellung gg. eMHSs,  Kurzskala, Post-Intervention | (1) E-Mental Health Angebote <*> können bei der Bewältigung von psychischen Problemen bzw. Stress und zur Stärkung von Resilienz von Nutzen sein.  (2) Ich würde E-Mental Health Angebote<*>, meinen Freunden/Bekannten, die psychische Probleme oder Stress haben, empfehlen. |
| Einstellung gg. Online-Therapien (klinisch) –APOI (Schröder et al., 2015), Post-Intervention | *Siehe: Schröder et al. (2015)* |
| Einstellung-gg, Online-Therapien (public health) - ETAM  (Apolinário-Hagen et al., 2018), Post-Intervention | (1) Internettherapien sind modern bzw. entsprechen unserer modernen Zeit.  (2) Internettherapien werden konventionelle Psychotherapien zukünftig ersetzen können.  (3) Internettherapien lassen sich besser mit Arbeit und Privatleben vereinbaren als konventionelle Psychotherapien.  (4) Es macht für mich keinen Unterschied, ob eine Psychotherapie über das Internet oder in der Praxis erfolgt.  (5) Internettherapien werden mehr Menschen mit psychischen Problemen erreichen.  (6) Krankenkassen sollten die Kosten für Internettherapien übernehmen.  (7) Internettherapien sind vergleichbar wirksam wie konventionelle Psychotherapien.  (8) Das Vertrauen zu einem Therapeuten/einer Therapeutin kann über das Internet genauso gut aufgebaut werden wie bei konventionellen Psychotherapien.  (9) Internettherapien sind eine geeignete Alternative zu konventionellen Psychotherapien.  (10) Bei psychischen Problemen würde ich eine Internettherapie in Anspruch nehmen.  (11) Ich würde eine Internettherapie einer konventionellen Psychotherapie vorziehen.  (12) Internettherapien werden mehr Patienten erreichen und ihnen helfen können.  (13) Ich mache mir keine besonderen Sorgen um den Datenschutz bei Internettherapien.  (14) Durch die Anonymität bei Internettherapien sinkt die Hemmschwelle, offen und ehrlich über wichtige Probleme zu sprechen.  (15) Durch die Verbreitung von Internettherapien werden sich Menschen früher professionelle Hilfe holen.  (16) Missverständnisse treten bei Internettherapien ähnlich häufig auf wie bei konventionellen Psychotherapien.  (17) Internettherapien eignen sich für die meisten Patienten, unabhängig vom persönlichen Hintergrund (Alter, Geschlecht, Bildung etc.). |
| Nutzungsabsicht – UTAUT (Hennemann et al., 2016), Post-Intervention | (1) Ich kann mir vorstellen, dass <**> mein Wohlbefinden fördern kann.  (2) Ich würde gerne ein Angebot <**> ausprobieren.  (3) Ich plane ein Angebot <**> in den nächsten 3 Monaten zu nutzen. |

**Anmerkungen**. Abkürzungen: eMHSs = eMental health services, APOI = Attitudes toward Online Interventions, ETAM = E-Therapy Attitudes Measure, IG = Interventionsgruppe, PSS = Perceived Stress Scale, IG = Interventionsgruppe, KG = Kontrollgruppe, UTAUT = Unified Theory of Acceptance and Use of Technology

<*> = Einsetzen eines Beispiels, je nach Gruppe: für IG1: „wie MH-Online“, für IG2: „wie GET.ON“, für IG3: „wie StudiCare“; für die KG: „ein solches Angebot“

**Anhang 3: Ergänzende Tabellen aus dem Ergebnisteil**

Tab. S1: Merkmale der Stichprobe (N=451)

| **Variablen** | | **Anteil, n (% von N=451)** |
| --- | --- | --- |
| **Geschlecht** | Weiblich | 340 (75.4) |
|  | Männlich | 110 (24.4) |
|  | Anderes/divers | 1 (0.2) |
| **Alter (Jahre)** | M (SD),  Median, Range (Jahre) | 32.6 (10.29),  29.00, 18-65 Jahre |
| **Bildungsabschluss** | Fachoberschulreife  Meisterbrief  Fachhochschulreife  Allgemeine Hochschulreife  Hochschulabschluss  - Bachelorabschluss  - Master-/Diplomabschluss  - Doktorgrad  Sonstige | 13 (2.9)  10 (2.2)  28 (6.2)  188 (41.7)  173 (38.3)  94 (20.8)  57 (12.6)  22 (4.9)  39 (8.6) |
| **Studienprogramm / Hochschulart** | Fernstudium / Fernuniversität | 400 (88.7) |
|  | Präsenzstudium / Präsenzhochschule^a^ | 27 (6.0) |
|  | Kombination aus Präsenz- und Fernstudium | 23 (5.1) |
|  | Anderes | 1 (0.2) |
| **Zeitmodell des Studiums** | Vollzeitstudium | 233 (51.7) |
|  | Teilzeitstudium | 217 (48.1) |
|  | Fehlende Angabe | 1 (0.2) |
| **Vertrautheit/ Bekanntheit von eMHSs^b^** | **Bekanntheit von eMHSs (Filterfrage)**  Nein  Ja  Weiß nicht  => n=227 Ja oder weiß nicht (50.3%) | N=451  224 (49.7)  191 (42.4)  36 (8.0) |
|  | **Bekanntheit von eMHSs = ja oder weiß nicht** | **Anteil, n (% von N=227)** |
|  | **Informationen über eMHSs gesucht**  Nein  Ja  Weiß nicht | n=227 / % von N=451  142 (62.6) / 31.5%  75 (33.0) / 16.6%  10 (4.4); 2.2% |
|  | **Erfahrung mit der Nutzung von eMHSs**  Nein  Ja  Weiß nicht | n=227 / % von N=451  183 (80.6) / 40.6%  32 (14.1) / 7.1%  12 (5.3) / 2.7% |

Anmerkungen. Abkürzungen: M= Mittelwert, SD= Standardabweichung, eMHSs= eMental health services

^a^ Traditionelle Präsenzhochschule in Abgrenzung zu Fernhochschulen (Durchführung der Studie von November 2018 bis Mai 2019, d.h. vor dem Ausbruch der Covid-19-Pandemie in Deutschland)

| Tab. S2: Deskriptive Daten, und Unterschiede zwischen und innerhalb der Gruppen bezüglich der Akzeptanz der eMHSs bei Studierenden (N=451) | | | | | | | | |
| --- | --- | --- | --- | --- | --- | --- | --- | --- |
| **Skala / Konstrukt** | **Range Skala**  **(M: min-max)** | **Insgesamt**  **(n=451)**  **M (SD)** | **KG**  **(n=112)**  **M (SD)** | **IG 1**  **MH-O**  **(n=115)**  **M (SD)** | **IG 2**  **GET.ON**  **(n=116)**  **M (SD)** | **IG 3**  **StudiCare**  **(n=108)**  **M (SD)** | **Between-Group^a^,**  **Effektstärke** | **Post-Hoc-Tests^b^** |
| Pre-Messung Nutzungsabsicht (Pre-Akzeptanz) | 1-7  (1.00-7.00) | 4.44  (1.12) | 4.42  (1.22) | 4.43  (1.12) | 4.48  (1.16) | 4.42  (0.97) | *F*_(3.447)_ = 0.10,  *p =*.972, ŋ_p_^2^=.001 | N.A. (n.s.) |
| Post-Messung Nutzungsab-sicht (Post-Akzeptanz) | 1-7.0  (1.00-7.00) | 4.28  (1.26) | 4.33  (1.29) | 4.27  (1.26) | 4.09  (1.31) | 4.43  (1.15) | *F*_(3.447)_ = 1.45, *p*=.227, ŋ_p_^2^=.01 | N.A. (n.s.) |
| Wahrgenom-mener Stress – PSS-10 (10 Items), Gesamt-Score | 10-50  (12-  47) | 27.04 (6.64) | 26.88 (6.64) | 26.88  (7.15) | 26.91 (6.28) | 27.53  (6.54) | *F*_(3.447)_ = 0.25, *p*=.860, ŋ_p_^2^=.002 | N.A. (n.s.) |
| Wahrgenommene Ähnlichkeit (5 Items, nur IGs, n=339) | 1-7  (1.00-7.00) | 3.95  (1.12)  (n=339) | N.A. | 3.87  (0.89) | 3.48  (1.15) | 4.54  (1.12) | *F*_(2.336)_ = 29.21, *p*<.001, ŋ_p_^2^= .15 (starke ES) | IG3 höher als IG1+IG2, und IG1 höher als IG2^b^  (*ps*<.05) |
| Glaubwürdigkeit (4 Items, insgesamt)^d^ | 1-7  (1.00-7.00) | 5.05  (1.00) | 5.15  (1.14) | 4.79  (1.07) | 5.22  (0.85) | 5.06  (0.88) | *F*_(3.447)_ = 4.07, *p*=.007,  ŋ_p_^2^=.03 (kleine ES) | IG2 höher als IG1^b^ (*p*=.005) |
| Glaubwürdigkeit der Informationen (2 Items)^d^ | 1-7  (1.00-7.00) | 5.08  (1.05) | 5.15  (1.14) | 4.86  (1.13) | 5.25  (0.91) | 5.07  (0.98) | *F*_(3.447)_ = 2.99, *p*=.031,  ŋ_p_^2^=.02 (kleine ES) | IG2 höher als IG1^c^, (*p*=.021) |
| Glaubwürdigkeit der Testimonial-quelle (2 Items, IG1-IG3, n=339)^d^ | 1-7  (1.00-7.00) | 4.98  (1.02)  (n=339) | N.A. | 4.73  (1.12) | 5.18  (0.95) | 5.05  (0.93) | *F*_(3.336)_ = 6.12, *p*=.002,  ŋ_p_^2^=.04 (kleine ES) | IG2 höher als IG1^c^, (*p*=.002), IG3 höher als IG1^c^ (*p*=.002), |
| Einstellung gegenüber eMHSs (Post-Messung), 2 Items | 1-7  (1.00-7.00) | 5.14  (1.13) | 5.06  (1.27) | 5.08  (1.17) | 5.24  (1.01) | 5.16  (1.08) | *F*_(3.447)_ = 0.58, *p*=.628, ŋ_p_^2^=.004 | N.A. (n.s.) |
| Einstellung gegenüber Psychologischen Online-Interventionen (APOI – Post-Messung), 16 Items | 1-5  (1.50-4.75) | 3.13  (0.52) | 3.03 (0.58) | 3.07  (0.54) | 3.21  (0.45) | 3.21  (0.50) | *F*_(3.447)_ = 3.46, *p*=.016, ŋ_p_^2^=.02 | Positiver in IG2 and IG3 als in IG1 und KG^d^ (*p_s_*<0.05) |
| Einstellung gegenüber Online-Therapien (ETAM – Post-Messung), 17 Items | 1-5  (1.35-4.71) | 3.08 (0.56) | 3.03 (0.62) | 3.02 (0.57) | 3.16 (0.51) | 3.10 (0.50) | *F*_(3.447)_ = 1.57, *p*=.197, ŋ_p_^2^=.01 | N.A. (n.s.) |

*Anmerkungen*. N=451. Abkürzungen: eMHSs = eMental health services, APOI = Attitudes toward Online Interventions, ETAM = E-Therapy Attitudes Measure, PSS = Perceived Stress Scale, ES = Effektstärke, IG = Interventionsgruppe, KG = Kontrollgruppe, M = (arithmetic) mean, M: min-max = Mittelwert: Mininum – Maximum, Mittelwert, SD = standard deviation, MH-O = MH-Online, N.A. = nicht anwendbar, n.s. = nicht signifikant,; Die Skalierung beim PSS-10-Summenscore wurde angepasst (Skalierung von 1-5 anstatt von 0-4). Die Daten in dieser Tabelle finden sich zum Teil auch in der Publikation zur Primäranalyse. Allen vier Versuchsgruppen wurden zwei Items zur Glaubwürdigkeit der Informationen vorgelegt, während die IGs zwei zusätzliche Items zu den Testimonials erhielten

^a^Einfaktorielle Varianzanalyse

^b^Games-Howell Post-hoc-Test

^c^Tuckey-HSD Post-hoc-Test

^d^Fisher’s LSD Post-hoc-Test
